# Cellular Uptake of Tau Aggregates Triggers Disulfide Bond Formation in Four-Repeat Tau Monomers

**DOI:** 10.1021/acschemneuro.4c00607

**Published:** 2024-12-23

**Authors:** Brad J. Krzesinski, Tyler J. Holub, Zachariah Y. Gabani, Martin Margittai

**Affiliations:** Department of Chemistry and Biochemistry, University of Denver, Denver, Colorado 80208, United States

**Keywords:** aggregation, Alzheimer’s disease, amyloid, conformation, disulfide, fibril, redox switch, seeding barrier, Tau protein, thiol

## Abstract

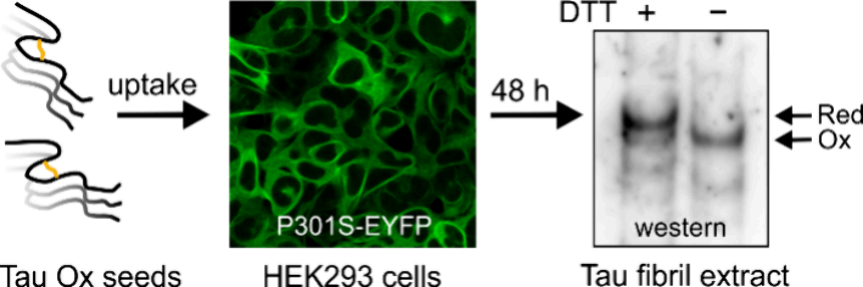

Oxidative stress is an important driver of aging and has been linked to numerous neurodegenerative disorders, including Alzheimer’s disease. A key pathological hallmark of Alzheimer’s are filamentous inclusions made of the microtubule associated protein Tau. Based on alternative splicing, Tau protein can feature either three or four microtubule binding repeats. Distinctively, three-repeat Tau contains a single cysteine; four-repeat Tau contains two. Although there is evidence that the cysteines in pathological Tau filaments exist in the reduced form, very little is known about the alternative disulfide-bonded state. It is unclear whether it can exist nontransiently in the reducing environment of the cytosol. Such knowledge, however, is important as different redox states of Tau could modulate aggregation. To address this question, we transfected HEK293 cells expressing the P301S variant of four-repeat Tau with fibril seeds composed of compact, disulfide-bonded Tau monomers. In vitro, these fibrils are observed to recruit only compact Tau, but not Tau in which the cysteines are reduced or replaced by alanines or serines. In line with this characteristic, the fibrils dissociate when treated with a reducing agent. When offered to HEK293 cells, variant Tau protein is recruited to the seeds forming intracellular fibrils with the same seeding properties as the in vitro counterparts. Markedly, the proteins in these fibrils have a compact, disulfide-bonded configuration and dissociate upon reduction. These findings reveal that uptake of exogeneous fibril seeds triggers oxidation of Tau monomers, modulating intracellular aggregation.

## Introduction

Filamentous inclusions composed of the microtubule associated protein Tau are a pathological hallmark of Alzheimer’s disease and over 20 other fatal neurodegenerative disorders, collectively known as tauopathies.^1−3^ There are multiple lines of evidence indicating that Tau pathology spreads throughout the brain via a prion-like mechanism whereby small Tau fibrils (or oligomers) transfer from one neuron to another and then recruit endogenous Tau monomers onto the fibril ends.^4,5^ The fibril serves as a template that forces the incoming intrinsically disordered monomer into its fold, with identical residues in different Tau monomers being stacked on top of each other and β-strands expanding perpendicular to the long fibril axis.^6^ The propagation of Tau fibrils throughout the brain is thought to be facilitated by fracturing into smaller aggregates that possess a higher mobility and seeding competency than the larger fibrils.^7^ Recent findings suggest that seeding-competent Tau species can be generated from fibrils via disaggregation by both the Hsp70 (heat shock protein 70) and the VCP (valosin-containing protein) chaperone systems.^8,9^

In the adult human brain six Tau isoforms are generated by alternative splicing, resulting in proteins ranging from 352 to 441 amino acids in size. The isoforms are distinguished by either three or four microtubule binding repeats in the C-terminal half of the protein and zero, one, or two inserts in the N-terminal region. Based on the number of microtubule binding repeats, Tau is categorized as either three-repeat (3R) Tau or four-repeat (4R) Tau. The repeat region makes up most of the protease resistant core of Tau fibrils^10,11^ whereas the flanking regions form a highly disordered fuzzy coat.^12,13^ Alzheimer’s disease is a mixed tauopathy in which all Tau isoforms are deposited into fibrils.^14,15^ In other tauopathies such as progressive supranuclear palsy, corticobasal degeneration, and argyrophilic grain disease, fibrils are composed of solely 4R Tau isoforms.^16−18^ Fibrils in Pick’s disease, on the contrary, contain solely 3R Tau.^19^ The preferential deposition of isoforms in these tauopathies can be explained by structural incompatibilities between fibril seeds and monomers, which disallows the recruitment of 3R tau monomers onto 4R Tau seeds and vice versa.^20,21^ Recent cryo-EM data have revealed unique fibril folds for different tauopathies.^22^ Markedly, even fibrils composed of the same types of isoforms can differ in structure.^23^ The molecular basis for this conformational diversity is poorly understood, although posttranslational modifications that alter the charge density in Tau could play a critical role.^24^

One modification that has received early attention is the oxidation of cysteines to cystines.^25^ It has been postulated that in the aging brain Tau dimers could nucleate Tau fibril formation under oxidative stress.^26^ There is accumulating evidence that a disruption in redox homeostasis is covaried with neurodegeneration.^27^ However, the molecular mechanisms remain largely unknown. 3R Tau harbors a single cysteine at position 322 in the third microtubule binding repeat. The formation of an intermolecular disulfide bond between Tau molecules has been observed to accelerate aggregation.^25,28,29^ 4R Tau contains two cysteines: one at position 322 in the third repeat and another one at position 291 in the second repeat. The presence of two cysteines leads to the formation of either inter- or intramolecular disulfide bonds. 4R Tau monomers with an intramolecular disulfide bond assume a more compact structure than 4R Tau monomers lacking this bond. There are several studies suggesting that compact Tau monomers are refractory to aggregation.^25,28,30^ Contrary to these findings Furukawa et al.^29^ observed that compact Tau monomers are aggregation-competent, forming a fibril polymorph with a smaller core size. We recently corroborated the formation of fibrils from these compact 4R Tau monomers and elucidated that the disulfide bond serves as a reversible redox switch that controls the assembly and disassembly of the fibrils.^31^ Notably, Tau monomers without an intramolecular disulfide bond were not recruited into these fibrils, delineating clear barriers of seeding. Although none of the pathological Tau fibrils solved to date show any disulfide bonds, it is possible that the redox switch could play an important role in segregating Tau into different pools.^31^ However, since the redox switch has only been observed under controlled in vitro conditions, a key question remaining is whether this switch can also exist in cells. After all, the cytosol where Tau aggregation occurs is a reducing environment, with millimolar concentrations of glutathione.^32,33^ It is not clear whether oxidative stress is sufficient to measurably alter the pools of Tau species.

Here we set out to address this question using Tau fibrils composed of compact Tau monomers as novel model system. Our study reveals that these fibrils when offered to HEK293 cells initiate Tau aggregation within the cells. The cellular aggregates exhibit the same seeding properties as the transfected fibrils and are also composed of compact Tau, indicating that reduced Tau monomers in the cells have been converted into the disulfide-bonded form.

## Results

### Compact Tau Monomers Assemble into Fibrils That Exhibit Homotypic Seeding

**Figure 1:**
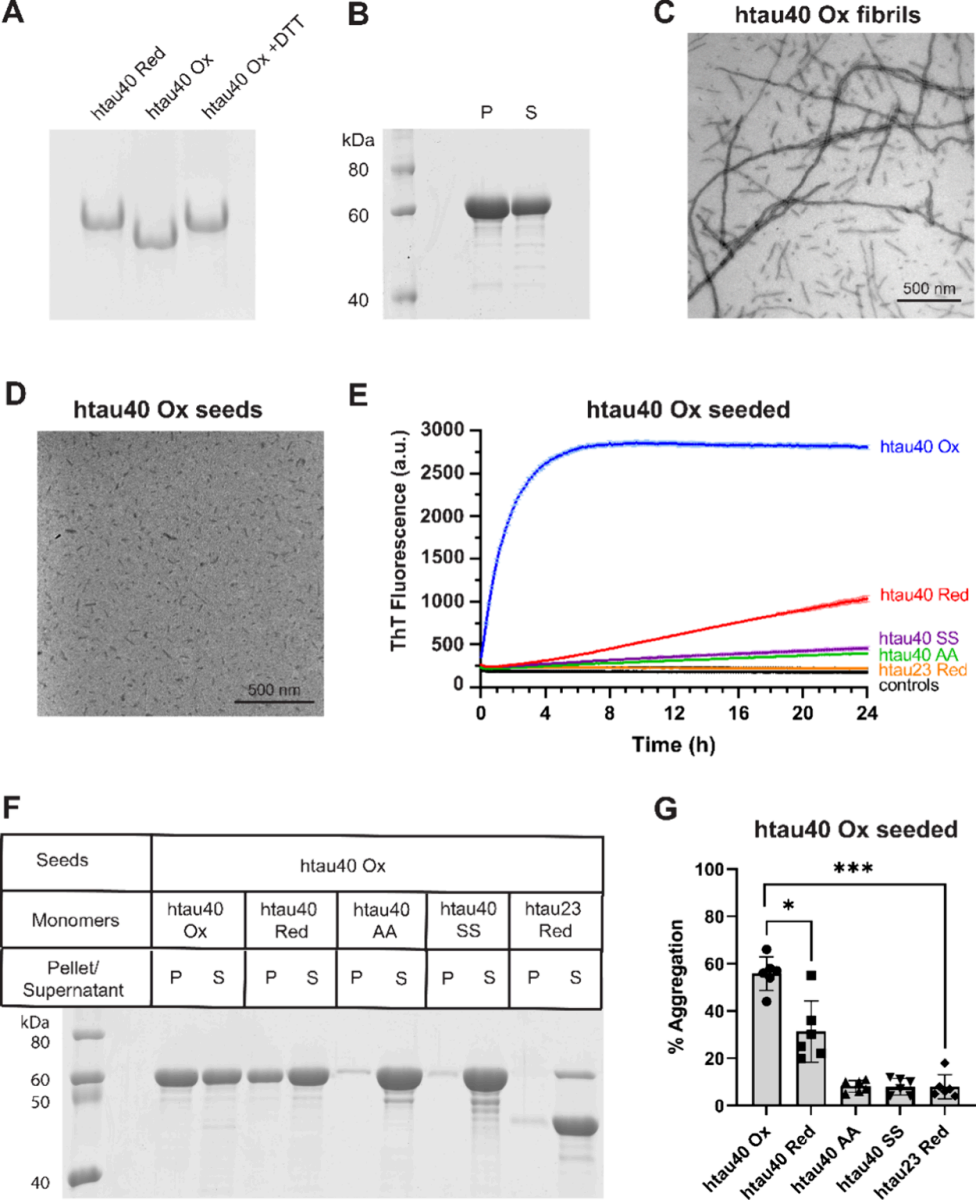
Fibril seeds composed of htau40 Ox recruit homotypic but not heterotypic Tau monomers. (A) Formation of compact Tau monomers was assessed by native, nonreducing PAGE. (B) Fibrils were assembled in the presence of heparin, centrifuged, and analyzed by SDS-PAGE and Coomassie staining. Visualization of fibrils (C) and sonicated fibrils or seeds (D) by negative stain EM. (E) Kinetic analysis of fibril growth. Fibril seeds made from htau40 Ox were mixed with different Tau monomers (blue, htau40 Ox; red, htau40 Red; purple, htau40 SS; green, htau40 AA; orange, htau23 Red) and incubated for 24 h at 37 °C. ThT fluorescence was monitored over time. Seeds (htau40 Ox) and monomers by themselves (htau40 Ox, htau40 Red, htau40 SS, and htau40 AA) served as controls (black). All reactions were performed in triplicate. (F) Incorporation of monomers into Tau fibrils was separately examined by SDS-PAGE and Coomassie staining. For this purpose, the monomer/seed mixture was centrifuged after incubation and equivalent amounts of pellet and supernatant loaded onto a gel. (G) Quantitative analysis by gel densitometry. Statistical comparison was performed using paired *t* tests. *p = 0.015 and ***p < 0.001. Data from six experimental replicates. Error bars represent means ± SD. P, pellet; S, supernatant; ThT, thioflavin T.

Various oxidizing environments have been utilized to generate compact Tau monomers. They include the addition of Cu^2+,^^30,34^ hydrogen peroxide,^29,31^ and organic compounds such as methylene blue.^35−37^ Here, we employed atmospheric oxygen under mild basic conditions (pH 8.15) to form compact monomers of htau40 (also known as 2N4R). This created an intramolecular disulfide bond between the two native cysteines at positions 291 and 322. The compact structure of the protein was visibly differentiated by native gel electrophoresis as the oxidized monomer migrated faster through the gel than the reduced version (Figure 1A). Importantly, the addition of DTT to compact Tau monomers resulted in the same migration property as that of reduced Tau (Figure 1A), suggesting that the monomers had converted back into the extended configuration and that the reaction was reversible. To test the aggregation competency of compact Tau, the protein was incubated for 5–7 days at 37 °C in the presence of heparin, a negatively charged cofactor that facilitates fibril formation.^38,39^ Upon ultracentrifugation, the majority of Tau protein (∼60%) was found in the pellet (Figure 1B), indicating that the protein had assembled into larger aggregates. The fibrillar nature of these aggregates was verified by negative stain transmission electron microscopy (Figure 1C).

In the next set of experiments, we examined the seeding properties of the fibrils. For this purpose, the aggregates were first sonicated to generate smaller fragments also known as seeds (Figure 1D) and then mixed with different species of Tau monomers. Fibril elongation was monitored in the presence of Thioflavin T (Figure 1E), a dye that exhibits increased fluorescence upon binding to amyloid fibrils.^40^ Whereas compact Tau monomers rapidly added onto oxidized seeds, reduced monomers added much more slowly (Figure 1E). The delayed kinetics of latter monomers can be explained by the slow air-oxidation of the extended monomers into a compact, disulfide-linked form which is then homotypically recruited onto the oxidized seeds.^31^ Accordingly, Tau monomers in which the two native cysteines were replaced by alanines or serines exhibited even slower aggregation kinetics. For these variants there was only a marginal change in fluorescence intensity observed (Figure 1E). Similarly, monomers of the smallest Tau isoform, htau23 (also known as 0N3R), which lack the ability to form an intramolecular disulfide bond failed to assemble (Figure 1E). Importantly, in the absence of seeds, there was no spontaneous aggregation detected for any of the htau40 monomers (Figure 1E). Furthermore, when the seeds were incubated by themselves the ThT signal remained unchanged, indicating that the seeds did not undergo structural transformation during incubation. When the incubation time was increased from 24 to 48 h, the kinetic trends continued with slow seeded aggregation for htau40 Red, minor aggregation for htau40 SS and htau40 AA and no aggregation for htau23 or any of the monomer and seed controls (Figure S1).

Finally, we sought to validate the seeding properties of oxidized Tau fibrils using an alternative readout. The seeding experiments were repeated, but this time the samples were sedimented after incubation and analyzed by SDS-PAGE and Coomassie staining (Figure 1F). Quantification of the data (Figure 1G) revealed that seeding with oxidized fibrils was most efficient when oxidized monomers were offered (56% aggregation). Recruitment of reduced monomers was less efficient (31%) as these monomers first had to convert into the compact form. The double alanine and double serine variants of htau40, like htau23, failed to be recruited altogether (Figure 1F,G). Note that in these experiments unreacted seeds remain in the supernatant because of their small overall size,^41^ which was only apparent for the htau40-seeded reaction with htau23 as a substrate (Figure 1F). Together, the data indicate that short fibrils of air-oxidized htau40 recruit Tau monomers with a compact configuration but not Tau monomers with an open, extended configuration, corroborating the structural incompatibility between these two states. In line with these observations, fibrils composed of compact monomers (but not reduced monomers) dissociate when treated with DTT (Figure S2). The data are in agreement with the previously reported seeding properties and dissociation characteristics of oxidized htau40 fibrils^31^ and suggest that the generated Tau seeds are suitable for testing aggregation in cell culture.

### Oxidized Tau Seeds Induce Aggregate Formation in HEK293 Cells Overexpressing the Pathogenic P301S Mutant of Tau

Given that Tau monomers must contain an intramolecular disulfide bond to be recruited onto oxidized htau40 fibrils, the question arose whether these fibrils exhibit any seeding competency within the reducing environment of the cytoplasm. To address this question, we employed a monoclonal cell line of human embryonic kidney 293 (HEK293) cells that expresses the pathogenic P301S mutant of htau40 fused at the C-terminus with enhanced yellow fluorescence protein.^42^ This mutant was chosen because, in contrast to wildtype Tau, it readily aggregates in HEK293 cells when exogeneous seeds are offered.^43^

**Figure 2:**
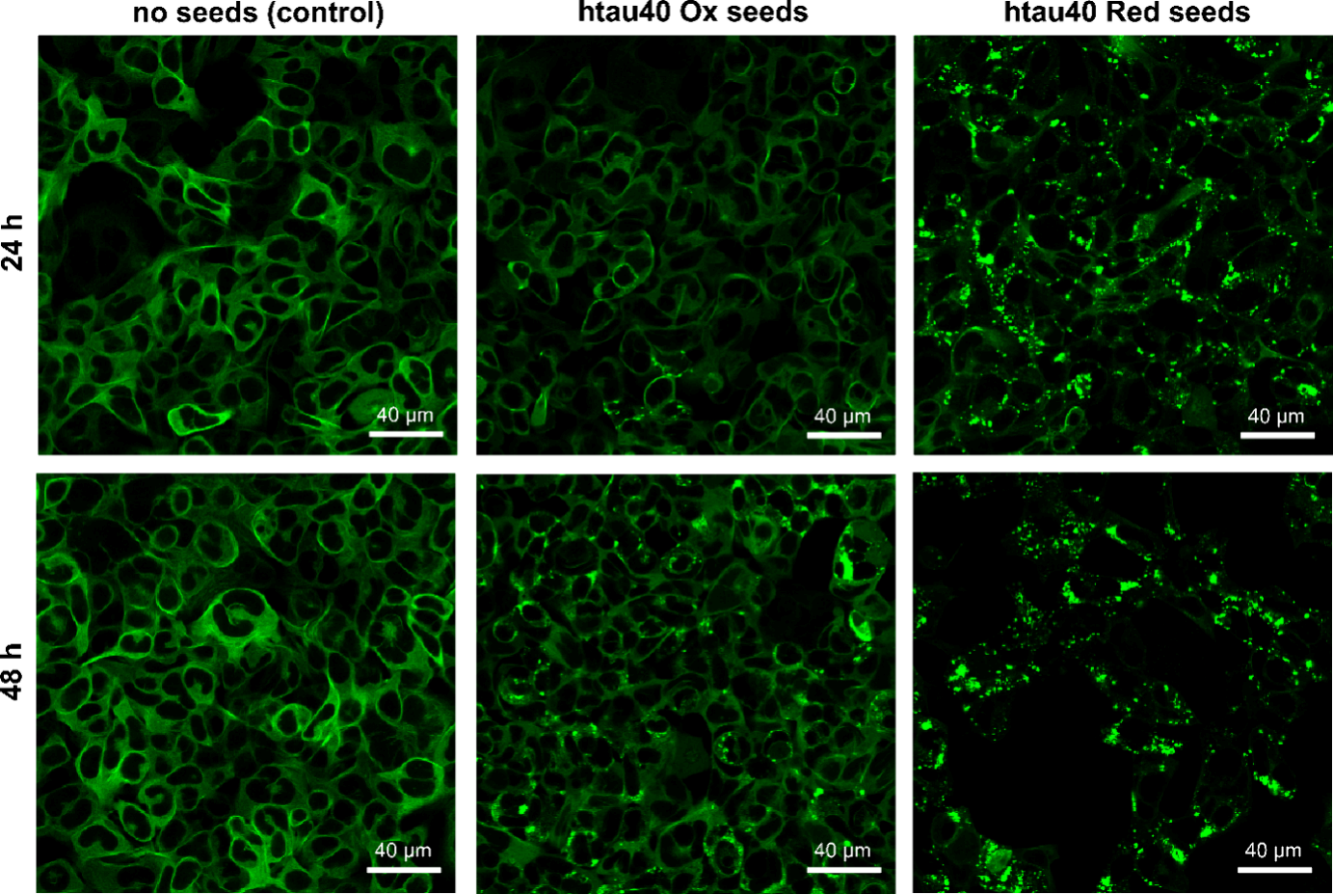
Oxidized and reduced htau40 seeds induce Tau aggregation in HEK293 cells. Monoclonal HEK293 cells expressing htau40P301S linked to EYFP were transfected with buffer (left), htau40 Ox seeds (center), or htau40 Red seeds (right), and imaged after 24 (top) and 48 h (bottom) of incubation.

In a first step, reduced and oxidized htau40 fibrils were generated in vitro as described above. The fibrils were then sheared by sonication and seeds offered to the cultured cells. The addition of buffer served as a control. Images were taken by confocal microscopy after 24 and 48 h (Figure 2). Imaging after 24 h revealed abundant puncta throughout the cytosol for cells incubated with reduced seeds (Figure 2). These puncta reflect the uptake of seeds and subsequent aggregation of fluorescently tagged P301S monomers. At this time point, only a few puncta were observed in cells incubated with oxidized seeds, and no puncta were observed in cells that served as controls. After 48 h, puncta were still in high abundance for cells incubated with reduced seeds, but there was a significant decrease in viable cells compared to cells incubated with oxidized seeds as judged by the MTT (3-(4,5-dimethylthiazol-2-yl)-2,5-diphenyltetrazolium bromide) colorimetric assay (Figure S3). More importantly, after 48 h cells that were exposed to oxidized seeds also displayed abundant puncta indicating that these oxidized seeds induced aggregation. Again, no puncta were observed for the control cells. Together, the data suggest that both reduced and oxidized Tau fibrils exhibit seeding competency in HEK293 cells, but that the seeding by oxidized fibrils has slower kinetics.

### Tau Aggregates in HEK293 Cells Display Similar Properties as In Vitro Aggregates

**Figure 3:**
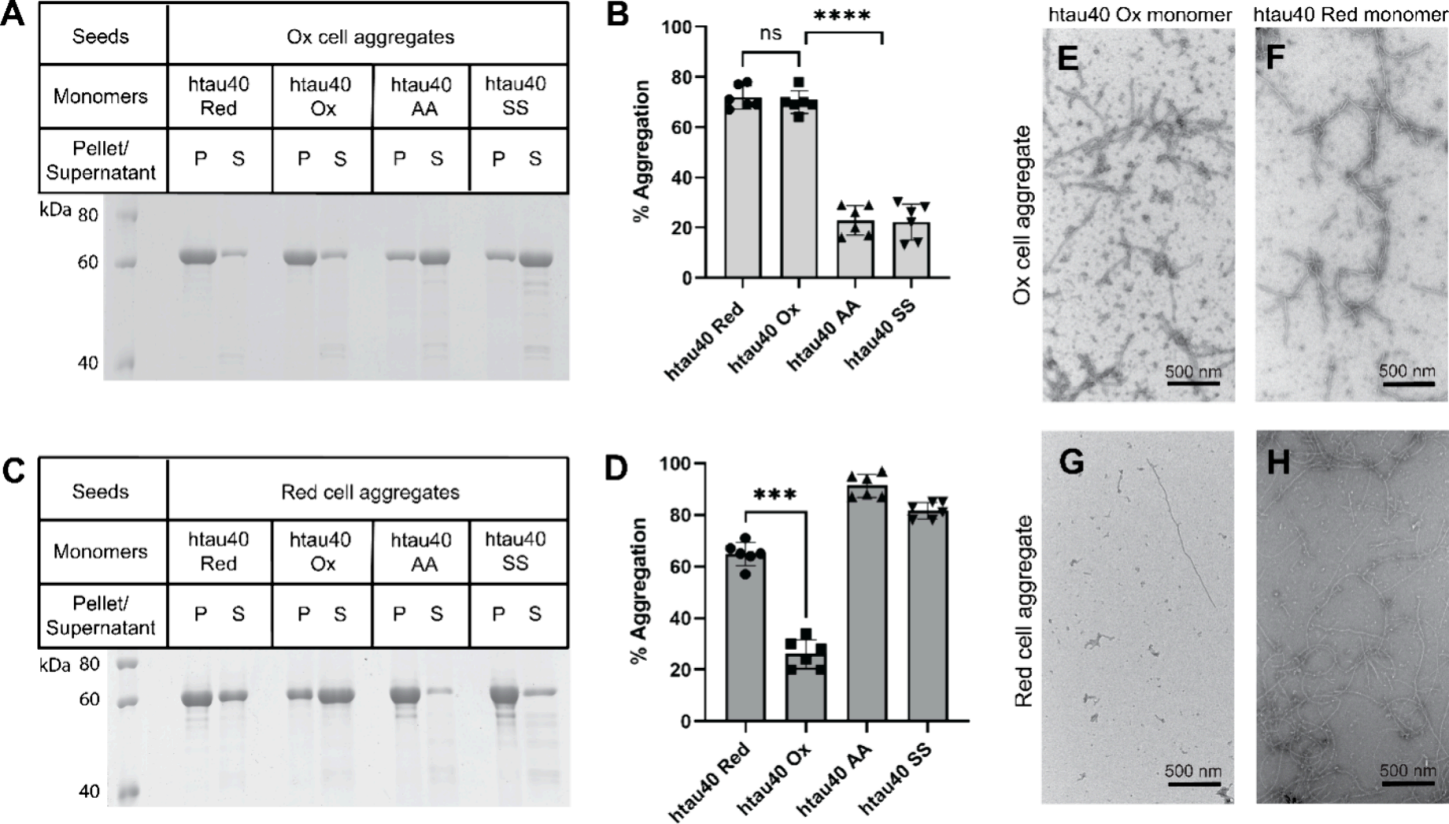
Tau aggregates isolated from HEK293 cells exhibit similar seeding properties as their in vitro counterparts. HEK293 cells expressing htau40P301S tagged with EYFP were transfected with htau40 Ox or htau40 Red seeds and cultured for 48 and 24 h, respectively. Cellular aggregates were extracted and centrifuged. Seeds were generated by sonication. Seeds from Ox cell aggregates were mixed with different htau40 monomers (htau40 Red, htau40 Ox, htau40 AA, or htau40 SS), incubated, centrifuged and analyzed by SDS-PAGE and Coomassie staining (A). Band intensities were quantified by densitometry (B). The analysis and quantifications were repeated for monomers seeded with Red cell aggregates (C, D). Statistical comparison was performed using paired *t* tests. ns = nonsignificant; ***p < 0.001; ****p < 0.0001. A total of six experimental replicates were collected. Error bars represent means ± SD. P, pellet; S, supernatant. (E–H) Negative stain transmission electron micrographs of htau40 Ox (E) and htau40 Red (F) monomers seeded with Ox cell aggregates. Micrographs of htau40 Ox (G) and htau40 Red (H) monomers seeded with Red cell aggregates.

One plausible explanation for the observed aggregation of Tau protein in HEK293 cells is that seeds, once internalized, recruit Tau monomers onto their ends. For oxidized Tau seeds this would mean that Tau monomers in the cell must first form an intramolecular disulfide bond. Such conversion would be consistent with the slower aggregation kinetics. An alternative explanation is that internalized seeds induce Tau aggregation via an indirect mechanism. Whereas a template-assisted process allows for propagation of the original fibril fold, a nontemplated process would be expected to cause a change in fibril structure. To distinguish between these two scenarios, we set out to characterize the seeding properties of the cellular Tau aggregates. First, the cells exposed to oxidized and reduced seeds were solubilized by a mild detergent (sarkosyl). Then, the cellular aggregates were harvested by ultracentrifugation. Seeds generated from these aggregates were combined with the same htau40 species that were used in the in vitro assays (Figure 1F,G) and incubated for 24 h. After sedimentation, the soluble and insoluble fractions were analyzed by SDS-PAGE. Seeds from cells treated with oxidized fibrils efficiently recruited reduced and oxidized htau40 monomers (∼71%), but not monomers in which the natural cysteines were substituted by alanines or serines (∼22%) (Figure 3A,B). The recruitment of reduced monomers onto the seeds could be explained by prior conversion of these monomers into the oxidized, compact form. Seeds from cells treated with reduced fibrils most efficiently recruited the double alanine (91%) and double serine (82%) variants of htau40 (Figure 3C,D). Reduced monomers were also recruited (65%) but oxidation of these monomers during incubation presumably lowered the efficiency of recruitment. The lowest efficiency was observed for oxidized monomers (26%) in agreement with the previously reported seeding barrier for this pair of species.^31^ Combined, the data suggest that the cellular aggregates retain the seeding properties of the in vitro species. To examine the shape of the aggregates, seeded reactions with reduced and oxidized monomers were next imaged by transmission electron microscopy. Fibrillar assemblies were visible in all micrographs (Figure 3E–H), suggesting that the original seeds in the cell extracts were able to elongate their structure.

### Cellular Fibrils Formed after Transfection with Oxidized Tau Seeds Are Composed of Oxidized Tau Monomers

**Figure 4:**
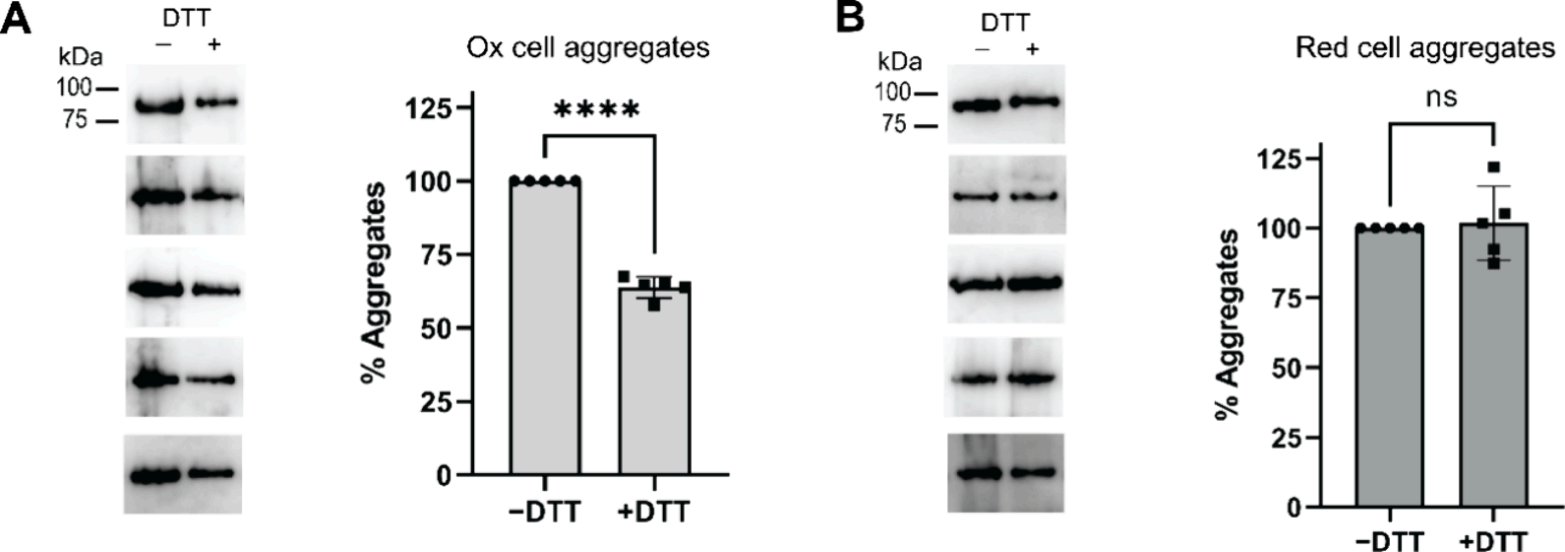
Cellular Tau aggregates formed upon transfection with oxidized Tau seeds partially dissociate when treated with DTT. Lysates of Tau-seeded HEK293 cells were treated with equal volumes of DTT or buffer, centrifuged, and processed by SDS-PAGE. Tau protein was detected after Western blotting using the Tau-5 antibody and quantified by densitometry. Statistical comparisons were performed using ratio paired *t* tests. Analysis and quantification of oxidized (A) and reduced cell aggregates (B). Five experimental replicates were collected. Error bars represent means ± SD. ****p < 0.0001; ns = nonsignificant.

Although the cellular fibrils exhibited similar seeding barriers as their in vitro formed counterparts, the oxidation status of the monomers within these fibrils remained unresolved. There was still no direct evidence that the Tau monomers possess an intramolecular disulfide bond. To shed light on this matter cell lysates containing the two different types of Tau fibrils (exogenously induced by oxidized and reduced seeds) were incubated in the presence or absence of 100 mM DTT. The samples were sedimented by ultracentrifugation and pellets analyzed by SDS-PAGE and Western blotting. It was found that the amount of Tau aggregates in cells transfected with oxidized seeds was significantly decreased (by >30%) when the lysates were treated with DTT compared to untreated samples (Figure 4A). Conversely, for cells transfected with reduced seeds there was no statistical difference in the amounts of aggregates regardless of whether the lysates were treated with DTT or not (Figure 4B). The findings suggest that former aggregates contained compact Tau monomers that dissociated upon reduction. Furthermore, it can be excluded that the effects are due to residual protein stemming from the originally offered exogenous seeds since the detected monomers matched the size of htau40 with an added EYFP tag. This tag is absent in Tau monomers composing exogenous seeds.

**Figure 5:**
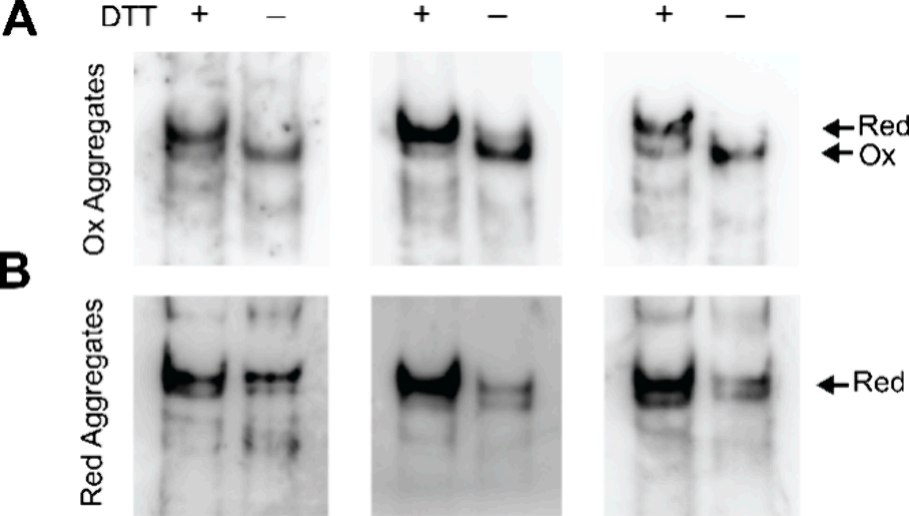
Cellular Tau aggregates formed after transfection with oxidized Tau seeds are composed of compact Tau monomers. HEK293 cells containing Tau aggregates were lysed with 1% sarkosyl, centrifuged, and taken up in urea with or without DTT. The samples were loaded and run on native, nonreducing gels and Tau protein detected by immunoblotting using the Tau-5 antibody. Tau protein from cell aggregates formed after transfection with oxidized Tau seeds (A) and reduced Tau seeds (B). Experiments were carried out in triplicate. Arrows highlight extended (Red) versus compact (Ox) structures.

Since the cellular aggregates did not dissociate completely upon reduction it was unclear whether they were composed solely of compact Tau monomers or whether they existed as a mixture of reduced and oxidized forms. To distinguish between these cases, compact monomers had to be separated from noncompact species. For this purpose, the cells were solubilized again in the absence of DTT, and aggregates were sedimented by centrifugation. To monomerize the fibrils, the pellets were next reconstituted in 8 M urea in the presence or absence of 100 mM DTT. The proteins were separated by native gel electrophoresis and blotted onto a membrane. Immunodetection revealed that Tau monomers from cells with oxidized Tau aggregates exhibited a downward shift compared to equivalent samples that were treated with DTT (Figure 5A). This shift is not observed for Tau monomers from cells with reduced Tau aggregates (Figure 5B). Here, both the DTT-treated and the nontreated proteins migrated the same. The data suggest that the cellular aggregates induced by oxidized Tau seeds are made exclusively of compact Tau monomers. A conversion of Tau monomers into the compact, oxidized form during urea reconstitution can be excluded since this would have also resulted in a band shift for the reduced fibrils, which was not observed.

## Discussion

Oxidative stress has been implicated in Alzheimer’s disease and other neurodegenerative disorders,^44,45^ however, its effects on the cysteine chemistry of Tau protein remain poorly understood. In this work we set out to gain first insights into the oxidation status of Tau in HEK293 cells using in vitro-formed fibrils made from compact air-oxidized htau40 monomers as exogeneous seeds. These fibrils are unique in that they selectively recruit Tau monomers with an intramolecular disulfide bond, but not Tau monomers with a single cysteine (htau23), two reduced cysteines (htau40), or monomers in which both cysteines are replaced by alanines or serines. Accordingly, the fibrils partially dissociated when treated with reducing agent as the compact configuration of Tau converted into an open extended state. These fibril properties are in agreement with those described in our previous study, in which Tau monomers were oxidized by either H_2_O_2_ or Cu^2+^, instead of air.^31^ More importantly, when the herein generated fibrils were offered to HEK293 cells expressing the P301S variant of Tau, the variant monomers were not only recruited, but the newly formed fibrils extracted from these cells also exhibited the same seeding properties as their in vitro counterparts. Notably, the cell-derived fibrils were composed entirely of compact monomers and partially dissociated when treated with DTT. These findings suggest that after the uptake of Tau seeds, variant Tau monomers in the cell must have converted from a reduced state that contains free thiol groups to an oxidized form that harbors an intramolecular disulfide bond. But what triggers the oxidation of Tau monomers to a compact disulfide-bonded state?

**Figure 6:**
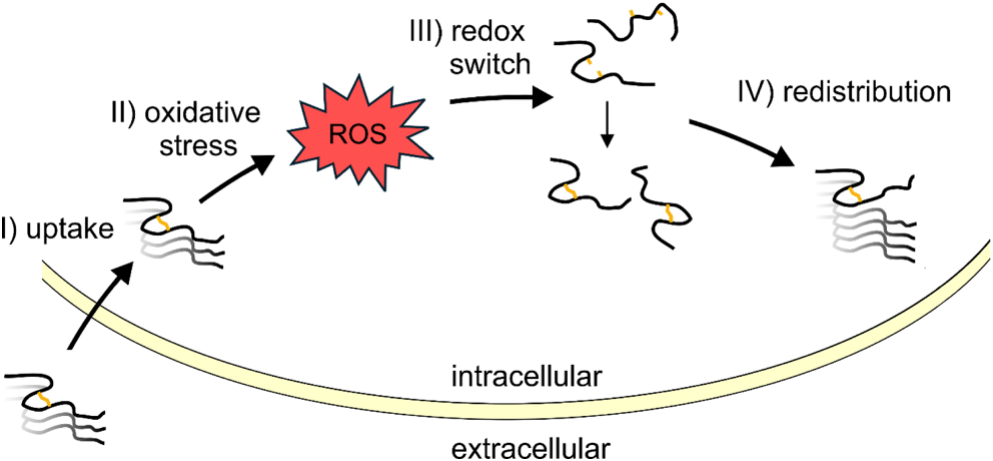
Intracellular Tau aggregates trigger a redox switch in four-repeat Tau monomers. Cellular uptake of Tau aggregates (I) results in oxidative stress (II) causing the production of reactive oxygen species (ROS). Tau monomers then switch from the reduced thiol form to the oxidized disulfide state (III). This causes a redistribution of Tau monomers into the fibril pool (IV). Note that this type of redistribution is only possible if the monomers are structurally compatible with the fibril seeds.

In mammalian cells disulfide bond formation in proteins is generally observed in the endoplasmic reticulum, the Golgi apparatus, and the intermembrane space of mitochondria.^46^ The cytosol in which Tau is localized features a reducing environment, sustained by a redox buffer with a high glutathione concentration^32,33^ that retains cysteines in their reduced state. However, there are exceptions to the general rule that proteins in the cytosol are free of disulfide bonds. For one, cysteine pairing may occur transiently for catalytic purposes.^47^ Also, disulfide bonds can form as a result of metabolic changes^48^ or in response to oxidative stress.^49^ Notably, the cysteines in Tau protein have been observed to form transient disulfide bonds with cysteines in the α- and β-subunits of tubulin.^50^ Furthermore, disulfide bond formation in Tau protein has been linked to unconventional secretion in which Tau penetrates the plasma membrane.^51,52^ A recent study demonstrated disulfide bond formation in primary mouse neurons and fly retina that resulted in the stabilization and accumulation of Tau protein.^53^ Although the specific cysteine linkages remained unresolved, it was hypothesized that an intramolecular bond between cysteines 291 and 322 with an altered overall conformation contributed to the stabilization. Combined, these findings provide precedence for Tau protein being oxidized within the cytosol under both physiological and pathological conditions. Tau disease variants have been linked to the malfunction of mitochondria and the generation of reactive oxygen species in different tauopathy models.^54^ Although oxidative stress may be a trigger of Tau aggregation,^55−57^ there is also evidence that Tau aggregates themselves are a cause of oxidative stress.^58−60^ In the context of the current study, these observations lead us to propose a model in which internalized Tau seeds induce oxidative stress in the cytosol, triggering the conversion of reduced Tau monomers to compact disulfide-bonded species that are then recruited and trapped within the oxidized fibril (Figure 6). The cysteines within Tau monomers thus resemble a redox switch capable of sensing oxidative stress.

Typically, disulfide-based redox switches are reversible, executing different biological functions in their different structural states.^61^ In the current system, the conversion of Tau protein from the open to the compact form appears to be unidirectional because of the continued oxidative stress associated with the aggregation. It is not clear how Tau aggregation inside the cell triggers oxidative stress and what species are the main culprits, but it is conceivable that smaller Tau aggregates that are generated in the propagation process may have the largest effect. This would be in line with the observation that large Tau filaments are generally inert whereas short Tau fibrils and small oligomers are most toxic.^62^ The investigated system is special in that oxidized Tau fibrils sequester compact Tau monomers from the soluble pool. A similar sequestration is not expected for reduced Tau fibrils, given that these fibrils are incompatible with the recruitment of compact Tau monomers. In this case the disulfide-bonded monomers will remain in the cytosol, possibly forming alternative types of assemblies. Hence, the disulfide bond in Tau could segregate the protein into different pools. Accordingly, none of the pathological Tau fibrils that have been solved thus far harbors an intramolecular disulfide bond. Indeed, the two cysteines within these fibrils are pointing in different directions, lacking the required proximity for forming disulfide bonds.^23,63−65^ However, there is also evidence that fibril structure can evolve over time^66−68^ and that changes in the environment can alter the populations of fibril conformers.^69−71^ Therefore, oxidized Tau species could play an important role in the early stages of aggregation. Although much more research is needed, it is conceivable that in the human brain, Tau protein can switch between different redox states, affecting both its biological functions and its pathophysiological effects. Understanding the molecular factors that regulate this switch, and the biological consequences should provide important new insights into Tau biology and Tau-associated diseases including Alzheimer’s.

## Experimental Procedures

### DNA Constructs

DNA inserts coding for human wildtype htau40 and htau23 proteins were previously cloned into pET-28 vector using the Nco1 and Xho1 restriction sites.^72^ The htau40 construct served as a basis for substituting both native cysteines with either serines or alanines, generating the cysteine-free versions htau40 SS and htau40 AA, respectively.^31^

### Expression and Purification

All proteins were expressed and purified according to published procedures.^72,73^ In short, BL21 (DE3)-competent *E. coli* cells were transformed with the appropriate pET-28 vectors by heat shock and then incubated on LB (Miller) agar plates (50 μg/mL kanamycin). Individual colonies were picked and grown in LB medium (20 μg/mL kanamycin) at 37 °C with agitation for 15–17 h. The cultures were diluted 1:100 with new LB medium (20 μg/mL kanamycin) and grown under agitation at 37 °C until optical density (OD) at 600 nm reached 0.7–1.0. Protein expression was induced by adding 0.5 mM isopropyl β-d-1-thiogalactopyranoside (IPTG) followed by agitation at 37 °C for 3.5 h. Bacteria were sedimented and resuspended in buffer (1 mM EDTA, 50 mM β-mercaptoethanol, 500 mM NaCl, and 20 mM Pipes, pH 6.5). To initiate protein extraction, the cells were heated for 15–20 min at 80 °C and then tip sonicated on ice for 1 min at 50% power. Soluble Tau protein was separated from insoluble material by centrifugation at 15,000*g* for 30 min, followed by precipitation with ammonium sulfate (55–60% w/v) while mixing slowly at room temperature overnight. Proteins were sedimented at 20,000*g* for 10 min, resolubilized in 4 mM DTT, tip sonicated for 4 min on ice, syringe filtered (GxF/GHP 0.45 μm), and then loaded onto a monoS 10/100 GL column (GE Healthcare). Proteins were eluted with a linear NaCl gradient (50–1000 mM NaCl, 2 mM DTT, 2 mM EDTA, 20 mM PIPES, pH 6.5) and analyzed by SDS-PAGE and Coomassie staining. The SDS sample for loading the gels had following composition: 5% 2-mercaptoethanol, 10% sucrose, 1.5 mM bromophenol blue, 62.5 mM Tris at pH 6.5, and 4% SDS). Fractions containing the highest concentrations of Tau protein were pooled and further purified by size exclusion chromatography. For this purpose, proteins were loaded onto a Superdex 200 column (10/300 GL), eluted with a TRIS buffer (2 mM DTT, 1 mM EDTA, 20 mM TRIS, pH 7.4, and 100 mM NaCl). Fractions containing pure Tau protein were pooled and then precipitated with an equal volume of methanol/2 mM DTT overnight on ice. Protein samples were centrifuged for 10 min at 15,000*g*, washed with methanol/2 mM DTT, aliquoted, and stored at −80 °C until further use.

### Protein Solubilization and Oxidation of htau40

Precipitated proteins were solubilized in 8 M GdnHCl, loaded onto PD 10 columns, (GE Healthcare) and eluted with assembly buffer (40 mM HEPES, 100 mM NaCl, and 0.1 mM NaN_3_ pH 7.4). Absorbance was measured at 280 nm and protein concentration determined using the molar extinction coefficient of Tau.

Monomers were stored either frozen or on ice, with 0.5 mM TCEP added for making reduced fibrils. Oxidized Tau (htau40) was formed by following the same monomerizing steps as above with the assembly buffer set at pH 8.15 and no TCEP added. The protein was diluted to 20 μM and oxidized in air at 22 °C with minimal agitation for 1–3 weeks. Oxidized protein was spin concentrated for 30 min at 6000*g* (Vivaspin 20 Sartorius) and then loaded onto a Superdex 200 column (10/300 GL) to separate oligomers from compact monomers. Fractions were analyzed by nonreducing SDS-PAGE. Monomer fractions were combined, spin concentrated and precipitated with methanol on ice overnight. Protein pellets were stored at −80 °C.

### Native PAGE

The protocol for native PAGE was adapted from previously described procedures.^35,74^ Electrophoresis was carried out in a Mini-Protean III cell (Bio-Rad). Gels were made of 8% acrylamide (37.5:1 acrylamide/bis(acrylamide)) and buffer containing 20 mM lactic acid and 30 mM β-alanine adjusted to pH 3.9. The same buffer was also used for the lower and upper gel compartments. Proteins (5 μM dilutions) were mixed with 2.5× sample buffer (55 mM lactic acid, 25% glycerol, 0.01% methyl green, and 75 mM β-alanine) and loaded onto the gel. The gel was run for 105 min at 30 mA with polarity reversed and then stained with Coomassie blue.

### Seed Preparation

Oxidized Tau fibrils were formed by combining 25 μM compact htau40 monomers with 50 μM heparin (Celsus; average molecular weight of 4400 Da) in assembly buffer and incubating the mixture for 5–7 days at 37 °C under agitation. Reduced Tau fibrils were formed the same way except that 0.5 mM TCEP was included during incubation. To generate seeds, the fibrils (400 μL total volume) were tip-sonicated for 30 s om ice at 20% power using a Fisher Scientific sonifier (model 100 with a 2 mm tip).

### Seeded Reactions

Tau monomers (10 μM), mixed with 10% seeds (monomer equivalents) and 20 μM heparin, were incubated quiescently for 24 h at 37 °C. The samples were centrifuged for 30 min at 256,000*g* (10 °C) and separated into pellets and supernatants. The volumes were adjusted with SDS sample buffer to allow comparison of equivalent amounts by SDS-PAGE (12%) and Coomassie staining. The percentage of fibril formation was quantified by ImageJ software (National Institutes of Health). For this purpose, the band intensity of the pellet was divided by the total band intensity (pellet plus supernatant) and then multiplied by 100. GraphPad Prism 7 software was used for plotting the data.

### Fibril Dissociation

After 24 h of seeding, (above) reactions were split equally. To one portion, 100 mM DTT were added to the other portion, assembly buffer was added (to same volume). Reactions were incubated for another 24 h before being centrifuged for 30 min at 256,000*g* (10 °C). Pellets were separated from supernatants, mixed with sample buffer, and analyzed by SDS-PAGE and Coomassie staining.

### Thioflavin T Assay

To monitor fibril elongation in real time 10 μM Tau monomers were mixed with 10% seeds, 20 μM heparin, and 5 μM, Thioflavin T. The mixtures were incubated in a BGM Labtech FLUOstar Omega plate reader, quiescently at 37 °C for 48 h. Excitation wavelength was set to 440 nm while fluorescence emission was recorded at 480 nm. All readings were taken through the bottom of a 96-well optical PolymerBase plate (Thermo Scientific).

### Transmission Electron Microscopy

Samples for TEM evaluations were diluted to ∼1.5 μM. A 10 μL drop was placed onto Formvar/carbon-coated 200 mesh copper grids (Electron microscopy Sciences) for 45–90 s. The grid was then dabbed on filter paper to remove extra liquid followed by another 45–90 s incubation with a 10 μL drop of 2% uranyl acetate and dabbed on filter paper again. Grids were air-dried for 10–15 min. Images were collected on an FEI Tecnai T12 BioTwin electron microscope at 100 keV equipped with a Gatan CCD camera.

### Tau Aggregation in Cell Culture

Monoclonal htau40P301S-EYFP HEK293 cells^42^ were plated at 10,000–20,000 cells per well in a 96-well plate containing 10% FBS in DMEM and 700 μg/mL G418 (total volume of 210 μL). Cells were grown until they reached 70 to 100% confluency. Seeds composed of oxidized or reduced Tau were mixed with Lipofectamine 2000 and Opti-MEM and then added to cell media to achieve a final seed concentration of 1 μM. Buffer (100 mM NaCl, 10 mM HEPES, pH 7.4) plus Lipofectamine 2000 was used as a control. Cells were incubated and imaged after 24 and 48 h using an Olympus FluoView FV3000 Confocal Microscope equipped with a 488 nm laser. Experiments were carried out with three different seed batches.

### Properties of Cellular Tau Aggregates Determined by Western Blotting

Monoclonal htau40P301S-EYFP HEK293 cells were plated in a 6-well plate and seeded at 70–100% confluency as described above. After incubation with seeds (24 h with reduced fibrils and 48 h with oxidized fibrils), cells were washed with 1 mL PBS and then incubated for 2–3 min with 300 μL extraction buffer (Halt Protease Inhibitor Cocktail (Thermo Scientific), 1 mM EGTA, 5 mM EDTA, 10 mM TRIS-HCl, 150 mM NaCl and 1% sarkosyl). Extracts were combined and then sheared five times with a 27-gauge needle.

To determine the stability of cellular aggregates toward reducing agent, lysates were treated with either 100 mM DTT (in extraction buffer) or with extraction buffer (with no DTT) and then incubated for 2–4 h at 22 °C. Samples were centrifuged for 70 min at 258,000*g* (4 °C); pellets were resuspended in sample buffer, heated for 5–10 min at 96 °C, and loaded onto 12% SDS-PAGE gels. The proteins were blotted onto a PVDF membrane using a Semi-Dry Transfer Cell (Bio-Rad). The membrane was blocked for 50 min with 5% BSA/TBS (20 mM TRIS, 137 mM NaCl, 0.1% Tween 80 at pH 7.6), incubated for 1 h with Tau-5 antibody (Thermo Scientific; Cat# PIMA512808; 1:400 dilution), washed, incubated for 1 h with light chain binding protein conjugated to HRP (m-IgGκ; Santa Cruz, Cat# sc-516102; 1:1000 dilution), and washed again. Next, the membrane was exposed for 1 min to SuperSignal West Femto Maximum Sensitivity Substrate (Thermo Scientific) and then imaged with a Western blot imaging system (Azure Biosystems 600).

To determine the composition of cellular Tau aggregates (extended versus compact monomers), lysates were processed the same way as described above but with no DTT added prior to ultracentrifugation. Each respective cell lysate was divided into two equal aliquots for ultracentrifugation.

The aggregate pellets were resuspended in either 8 M Urea or 8 M Urea/100 mM DTT and then incubated for 4 h at 22 °C. Samples were loaded onto an 8% native gel, and proteins imaged after Western blotting.

### Recruitment of Recombinant Tau Monomers to Aggregates Isolated from HEK293 Cells

Pellets from cell lysates (above) were reconstituted in assembly buffer and sonicated for 1 min at 20% power on ice. Recombinant Tau monomers (10 μM; oxidized, reduced, AA, & SS) were mixed with heparin (20 μM), ThT (5 μM), and seeds (100 μL) and incubated quiescently at 37 °C for 24 h. The reactions were centrifuged at 258,000*g* for 30 min. Pellets and supernatants were taken up in sample buffer and evaluated by SDS-PAGE and Coomassie staining.

### MTT (3-(4,5-Dimethylthiazol-2-yl)-2,5-diphenyltetrazolium bromide) Assay

Monoclonal htau40P301S-EYFP HEK293 cells were grown and maintained in a 96-well plate and seeded at 70–100% confluency as described above. At the time points 24 and 48 h, cell culture medium was replaced with 100 μL of PBS and 10 μL of 5 mg/mL MTT. Cells were incubated for 1 h at 37 °C. After incubation, PBS with MTT was removed and 100 μL of DMSO per well was added. The plate was then shaken at 500 rpm in an Eppendorf Thermomixer C for 1 min followed by absorbance measurements at 540 nm in Biotek HT3 microtiter plate reader.

## Supporting Information Available

Data highlighting seeded Tau aggregation monitored by ThT fluorescence over 48h time window; partial dissociation of oxidized Tau fibrils in the presence of DTT; and cell viability assay (PDF)
