# Supplementary material for: Cellular Uptake of Tau Aggregates Triggers Disulfide Bond Formation in Four-Repeat Tau Monomers
Source: ACS Chem Neurosci. 2024 Dec 23;16(2):171–80. doi: 10.1021/acschemneuro.4c00607 (PMC11740991; doi:10.1021/acschemneuro.4c00607)
Supplement: Supplementary file 1 — cn4c00607_si_001.pdf [file cn4c00607_si_001.pdf]

SUPPORTING INFORMATION

Cellular Uptake of Tau Aggregates Triggers Disulfide Bond Formation in Four-Repeat Tau Monomers

Brad J. Krzesinski, Tyler J. Holub, Zachariah Y. Gabani, and Martin Margittai*

From the Department of Chemistry and Biochemistry, University of Denver, Denver, CO 80208, USA

*To whom correspondence should be addressed: Martin Margittai, Department of Chemistry and Biochemistry, University of Denver, 2190 East Iliff Ave, Denver CO 80208. Tel: (303)-871-4135; Fax: (303)-871-2254. E-mail: martin.margittai@du.edu

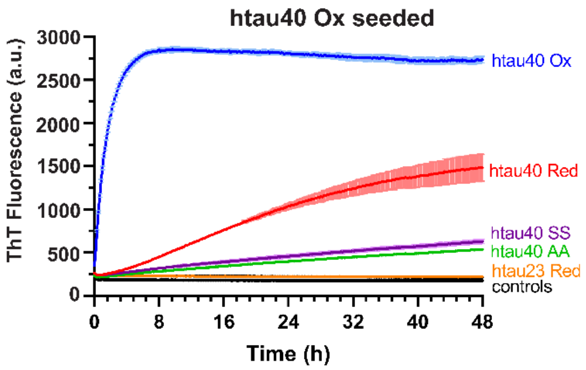

Figure S1. Fibril seeds composed of htau40 Ox effectively recruit homotypic, but not heterotypic Tau monomers. Tau seeds from htau40 Ox fibrils were generated by sonication, incubated at 37 °C in the presence of different Tau monomers and monitored by ThT fluorescence (blue, htau40 Ox; red, htau40 Red; purple, htau40 SS; green, htau40 AA; orange, htau23 Red). Htau40 Ox seeds and htau40 monomers (htau40 Ox, htau40 Red, htau40 SS, htau40 AA) in the absence of seeds served as controls (black). The graph captures an extended 48-h incubation window compared to the shorter 24-h window presented in Fig. 1E. All experiments were carried out in triplicate. Error bars represent means ± SD.

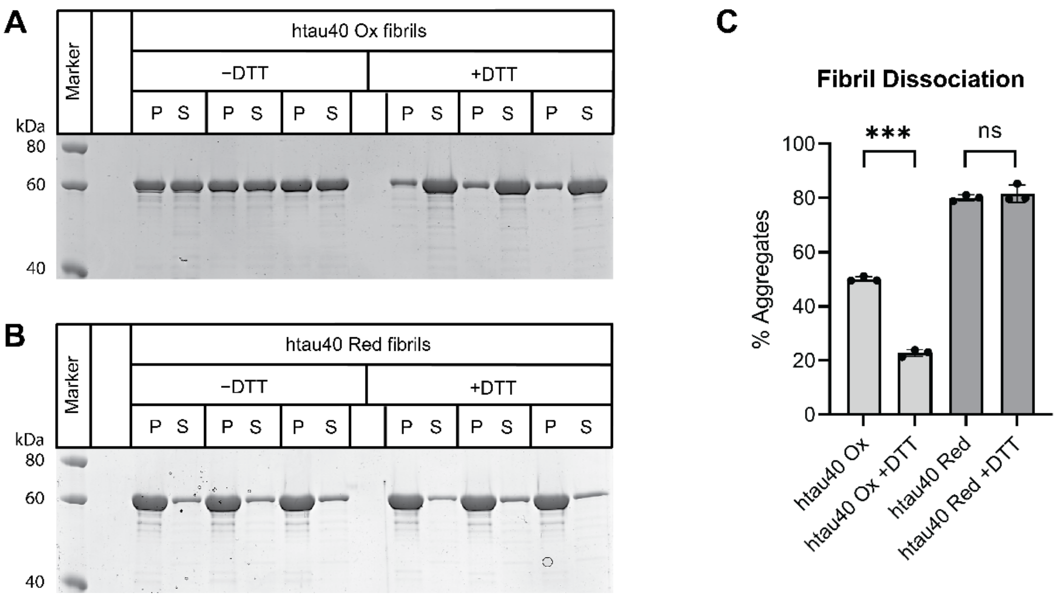

Figure S2. Tau fibrils composed of oxidized Tau monomers partially dissociate when treated with DTT. Tau seeds were combined with homotypic Tau monomers, incubated for 24 h to allow for elongation, and then treated with or without DTT. Aggregates were sedimented and then analyzed by SDS-PAGE and Coomassie staining. Analyses of htau40 Ox fibrils (A) and htau40 Red fibrils (B) are depicted. Experiments were carried out in triplicate. (C) Quantification and statistical analysis. Paired t tests were used for comparison of data. ***$p < 0.001$; ns = nonsignificant. Error bars represent means ± SD. P, pellet; S, supernatant.

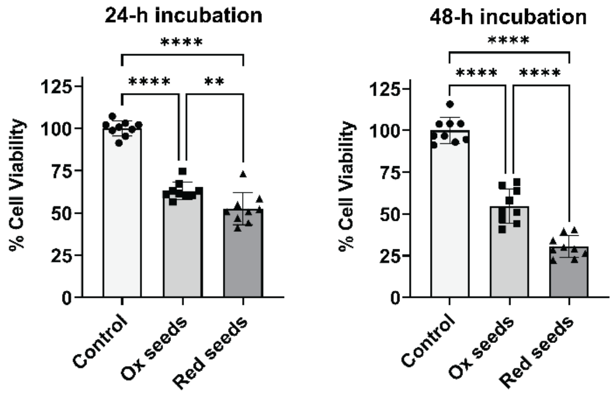

Figure S3. Tau puncta formation in HEK293 cells is associated with a decrease in cell viability. Monoclonal HEK293 cells expressing htau40P301S-EYFP were transfected with buffer, htau40 Ox seeds, or htau40 Red seeds, incubated for 24 h or 48 h at 37 °C, and then treated for 1 h with MTT. Absorbance was measured at 540 nm. MTT data of cells incubated for 24 h are shown on the left, MTT data for cells incubated for 48 h are shown on the right. Statistical comparisons were carried out using unpaired t tests. Nine replicates were collected for each condition. Error bars represent means ± SD. $^{**}p < 0.01$; $p^{****} < 0.0001$.
